# Current practices in marine systematic conservation planning: protocol for a global scoping review

**DOI:** 10.12688/openreseurope.16300.1

**Published:** 2023-09-05

**Authors:** Erika Fabbrizzi, Sylvaine Giakoumi, Dimitra Petza, Stelios Katsanevakis, Esther Domínguez Crisóstomo, Francesco Colloca, Michael Elliot, Wesley Flannery, Ibon Galparsoro, Maren Kruse, Emna Ben Lamine, Ben McAteer, Vanessa Stelzenmüller, Simonetta Fraschetti

**Affiliations:** 1 Department of Biology, University of Naples Federico II, Naples, Campania, Italy; 2 Sicily Marine Centre, Stazione Zoologica Anton Dohrn, Palermo, Sicily, 90149, Italy; 3 Department of Marine Sciences, The University of the Aegean, Mytilene, 81100, Greece; 4 Department of Biology, University of Algarve, Faro, Faro District, Portugal; 5 Integrative Marine Ecology Department, Stazione Zoologica Anton Dohrn, Rome, 00198, Italy; 6 Institute for Estuarine and Coastal Studies, University of Hull, Hull, England, UK; 7 Queen's University Belfast, Belfast, Northern Ireland, UK; 8 Marine Research Division, AZTI Foundation, Pasaia, Euskadi, 20110, Spain; 9 Thünen Institute of Sea Fisheries, Bremerhaven, 27572, Germany; 10 Universite Cote d'Azur, Nice, Provence-Alpes-Côte d'Azur, France

**Keywords:** Systematic Conservation Planning, Marine Protected Area networks, Ecosystem-Based Marine Spatial Management, climate change, connectivity, restoration, trade-offs analysis, conservation targets, scoping review protocol, JBI methodology, PRISMA statement

## Abstract

**Background**: Systematic Conservation Planning (SCP) involves a series of steps to identify conservation areas and develop management strategies, incorporating feedbacks, revisions, and iterations at any stage. It is a valuable tool in facilitating the effective implementation of Ecosystem-Based Marine Spatial Planning (EB-MSP). However, few efforts have been carried out to summarize information on methods, trends, and progress in SCP in the designation of Marine Protected Areas (MPAs). The present work aims at providing the protocol to perform a scoping review (ScR) to assess the contribution of SCP to the design of effective MPA networks, identifying both the development of good practices and the presence of gaps of knowledge in terms of criteria for their implementation.

**Protocol**: The ScR will follow the Joanna Briggs Institute (JBI) methodology. The Preferred Reporting Items for Systematic Reviews and Meta-Analyses (PRISMA) extension for ScRs supported the definition of this protocol. The three databases Web of Science, Scopus, and Google Scholar will be used for the bibliographic search. Inclusion criteria will be as follows: studies applying SCP in the marine realms worldwide, assessing its contribution to the design of MPA networks. Both peer-reviewed and grey literature will be considered for eligibility. No search limitations will be applied regarding publications’ year, stage, subject area and source type. Studies in English, French, German, Greek, Italian, and Spanish will be reviewed. Grey literature will be sourced from pre-print archives, institutional websites and other web-based search engines. The Covidence software will be used for the process of documents selection and data extraction. The findings of the ScR will be presented through tables, graphs, and maps, accompanied by a narrative summary of the outcomes.

**Conclusions**: This comprehensive approach will provide a visual representation of the data, enhancing the understanding and interpretation of the results.

## Introduction

Systematic Conservation Planning (SCP) is an approach involving a series of steps to identify conservation areas and develop management strategies, incorporating feedbacks, revisions, and iterations at any stage ( Kukkala & Moilanen, 2013). Previous experiences in terrestrial and coastal regions have shown that adopting a systematic approach to conservation planning and management can contribute to the preservation of ecosystem health and productivity ( Ban *et al.*, 2014). SCP enables effective and equitable management, aligning with various regional and global agreements focused on sustainable marine environment usage ( *e.g.*, CBD Aichi Target 11, SDGs, EU Biodiversity Strategy). There are several distinctive features associated with SCP. It requires a clear definition of the conservation target, including the selection of ecological features to protect or use as proxies for overall biodiversity within the planning process. It is built upon explicit operational objectives and assessing the progress made towards conservation goals through existing Marine Protected Areas (MPAs). SCP employs explicit methods for locating and designing new MPAs that complement existing ones to achieve the predefined goals, employing specific criteria for implementing conservation actions. Another important aspect of SCP is the principle of efficiency, *i.e.*, the explicit consideration of socioeconomic variables to minimize conflicts between conservation and existing (or planned) socioeconomic activities. Lastly, SCP adopts explicit objectives and mechanisms for maintaining conditions within MPAs that support the persistence of vital natural features, while also including monitoring these features for an adaptive management ( Margules & Pressey, 2000).

SCP can serve as a valuable tool in facilitating the effective implementation of Ecosystem-Based Marine Spatial Planning (EB-MSP). This management approach takes into account the complex interactions among ecosystem components and human activities at various spatial scales, rather than isolating individual sectors, species, or ecosystem services ( Halpern *et al.*, 2008; Leslie & McLeod, 2007). However, in this context, the designation of MPA networks is often based on structural characteristics of habitats and iconic species, rarely including the consideration of the ecological processes, thus disregarding the key SCP principles: connectivity, adequacy, representativeness, and efficiency. To address these issues, Katsanevakis *et al.* (2020) recommended SCP principles to ensure that MSP initiatives meet conservation requirements, establishing ecologically coherent networks of MPAs, and simultaneously address socioeconomic objectives ( *e.g.*, sustainable fisheries). Yet, only limited efforts have been made to consolidate information on methods, trends, and progress in SCP ( Álvarez-Romero *et al.*, 2018).

Here, we present a scoping review (ScR) protocol, aiming at assessing the contribution of SCP for designing MPA networks, providing an indication of the existence of successfully implemented marine plans, their impact on protecting biodiversity and identifying knowledge gaps.

The objectives of the ScR are as follows:

- Identify and critically evaluate the various methods and tools proposed for addressing key issues, such as (i) creating MPA networks resilient to climate change; (ii) securing marine functional connectivity; (iii) integrating terrestrial-freshwater-marine planning; (iv) embedding cumulative effects assessments in the planning process; (iv) assessing the potential Other Effective Conservation Measures (OECMs) for contributing to conservation targets; (v) including prioritization of habitats/species/areas for restoration, (vi) assessing synergies and trade-offs between passive and active restoration; (vii) addressing biological invasions; (viii) balancing conservation, socio-economic, and cultural objectives, and analysing trade-offs of planning options.
- Identify the gaps in current practices of SCPs for addressing these topics.
- Explore existing guidelines for improved conservation planning.

### Review question

The overall research question that will guide this ScR is: What is the current knowledge about Marine Systematic Conservation Planning approaches at a global scale? The ScR will attempt to answer the following sub-questions:

1. What is the geographical distribution of the case studies adopting SCPs?
2. Which are the criteria/methodologies/tools for implementing SCP, in particular, to address climatic resilience, functional connectivity, integrated land-freshwater-sea planning, cumulative effects, the contribution of OECMs, restoration site prioritization and actions, biological invasions, ecological-socioeconomic trade-offs?
3. What is the spatial contribution (% of the area covered) by case studies implementing SCP?
4. What are the limits in the adoption of good practices in SCP?
5. What are the success stories and failures acknowledged by the literature?
6. Which are the gaps of knowledge and policy recommendations provided by case studies implementing SCP?
7. What is the actual stage of implementation of SCPs?

## Methodology

The ScR will follow the methodology outlined by Arksey and O'Malley (2005), as further developed by Levac *et al.* (2010) and enhanced by the Joanna Briggs Institute (JBI). In particular, the methodology as defined Peters *et al.* (2020) for scoping reviews will be adopted.

The Preferred Reporting for Systematic Reviews and Meta-Analyses extension for scoping reviews, PRISMA-ScR ( Tricco *et al.*, 2018) supported the definition of this ScR protocol and will guide the final review paper. The PRISMA-ScR checklist, for a better understanding of relevant terminology, core concepts, and key items for scoping reviews, is available in the Open Science Framework repository at osf.io/e8h64.

The SUMARI Protocol Template for Scoping Reviews in Word format ( JBI SUMARI, 2021) has been used for the reporting of this ScR protocol.

### Inclusion/exclusion criteria

The development of the inclusion criteria for the ScR determining which sources will be considered for inclusion in the ScR, will be aligned with the mnemonic "Participants, Concept, and Context (PCC)" ( Table 1).

**Table 1.** Inclusion and exclusion criteria for the scoping review in correspondence with the "Participants, Concept and Context, PCC" mnemonic and evidence types and sources.

PCC and evidence types and sources	Inclusion criteria	Exclusion criteria
**P articipants** *Systematic conservation * *Planning (SCPs)*	Studies applying SCP (documenting specific implementation case studies or prioritization approaches/criteria/tools or including guidelines for SCP implementation or informing SCP approaches by providing software, scripts, and algorithms)	Studies that do not apply systematic conservation planning but other non- systematic approaches for selection of sites for protection
**C oncept** ***Methods and tools *** ***used for SCP***	All studies using SCP for designing Marine Protected Areas (MPAs), clearly describing the applied methods/ tools.	Studies that do not provide sufficient methodological details but simply mention the use of a specific SCP tool, *e.g.*, MARXAN.
**C ontext** *Global, marine realm*	Studies in: - Marine realm - Globally	Studies where the marine component includes only transitional systems. Studies without a marine component (primarily terrestrial or freshwater)
**Evidence types &** ** sources**	- Peer-review literature - Grey literature - All years of publication - All publication stages, subject areas, and source types - Experimental and observational studies - Case studies, reviews, framework/synthesis - Studies published in languages competent to the researchers’ team ( *e.g.*, English, French, German, Greek, Italian, Spanish according to the team's language competency)	---

***Participants***. The ScR will consider all studies applying SCP (documenting specific implementation case studies or prioritization approaches/criteria/tools or including guidelines for SCP implementation or informing SCP approaches by providing software, scripts, and algorithms). Studies applying other non-systematic approaches for the selection of sites for protection or restoration will not be taken into account.

***Concept***. The concept adopted by the ScR will be the assessment of how the contribution of SCP to the design of MPAs has been addressed, so far in the scientific literature. Studies that do not provide sufficient methodological details but simply mention the use of a specific SCP tool, *e.g.*, Marxan, will not be taken into account.

***Context***. Spatially explicit case studies in the marine realms worldwide will be considered by the ScR. Studies without a marine component (primarily terrestrial or freshwater) or where the marine component includes only transitional systems will not be taken into account.

***Types of sources***. This ScR will encompass peer-reviewed literature (such as research articles, reviews, book chapters, letters, editorials, books, and data papers) obtained from ISI (International Scientific Indexing) journal databases. Additionally, grey literature (including unpublished academic research, theses, policy papers and reports, conference abstracts and papers) will be sourced from pre-print archives, institutional websites and other web-based search engines. References suggested from topic experts and NGOs will also be taken into account. There will be no restrictions imposed regarding the publication year (the only cut-off will be set according to the date in which the literature searching is performed, see Table 2), publication stage (final or in press), subject area, or source type. All document types will be considered. Language restrictions will be implemented to reflect the language proficiency of the authors, including in the ScR only studies published in English, French, German, Greek, Italian, and Spanish.

**Table 2.** Details of scoping review search strategy per database, *i.e.*, name of database, date of search, search query, and results (as the number of documents returned by the search).

Database 1	Web of Science
**Date of ** **search:**	January 26, 2023
**Query:**	TS - "Systematic Conservation Plan*" **OR** "Marine Spatial Plan*" **OR** "Marine Zon*" **OR** Zoning **OR** "Marine Protected Area*" **OR** "Marine Reserve*" **OR** "Marine priorit*" **OR** "Marine planning" **OR** "Conservation priorit*" **OR** "Spatial optim*" **OR** "structured decision making" **OR** "Multi Criteria Decision Analysis" **OR** "multi criteria method" **OR** "cost effective* prioritization" **OR**"cost effective* prioritisation" **OR** "cost effective* analysis" **OR** "Spatial analysis" **AND** Marxan* **OR** MarCon* **OR** MarZone **OR** Spexan **OR** Spot **OR** Sites **OR** Zonation **OR** C-Plan **OR** CPLAN **OR** ResNet **OR** BioRap **OR** CREDOS **OR** MultCSync **OR** TARGET **OR** TRADER **OR** WorldMap **OR** prioritizR **OR** "Maritime Use Conflicts (MUC) Analysis" **OR** "Maritime Use Conflicts Analysis" **OR** "MarineMap" **OR** OceanMap **OR** CCRES **OR** CLUZ **OR** MinPatch **OR** "Zonae Cogito" **OR** "NatureServe Vista" **OR** PANDA **OR** SeaSketch **OR** PPGIS **OR** "Public Participation GIS" **OR** "Participatory GIS" **OR** PGIS **OR** GAMS **OR** CPLEX **OR** Gurobi **OR** "Integer Linear Program*" **OR** "integer programming" **OR** "multi-criteria" **OR** "multi-action" **OR** portfolio* **OR** "Decision Support System" **OR** DSS **OR** "decision support tools" **OR** DST **AND** "Protected area" **OR** "marine protected area" **OR** MPA **OR** "priority area" **OR** "area-based conservation" **OR** "conservation priorit*" **OR** "Management Area" **OR** "Nature Reserve" **OR** "Marine reserve" **OR** "National Park" **OR** Sanctuary **OR** "Marine Park" **AND** "Planning Unit*" **OR** "Analysis Unit*" **OR** cell **OR** hexagon* **OR** grid **OR** square* **OR** subcatchment* **OR** micro- catchment* **OR** "line segments" **OR** "linear segments" **OR** "linear units" **AND** marine **OR** sea* **OR** ocean* **OR** bay **OR** gulf **OR** coastal
**Results:**	**134** documents
**Database 2**	**Scopus**
**Date of** ** search:**	January 26, 2023
**Query:**	TITLE-ABS-KEY - "Systematic Conservation Plan*" **OR** "Marine Spatial Plan*" **OR** "Marine Zon*" **OR** Zoning **OR** "Marine Protected Area*" **OR** "Marine Reserve*" **OR** "Marine priorit*" **OR** "Marine planning" **OR** "Conservation priorit*" **OR** "Spatial optim*" **OR** "structured decision making" **OR** "Multi Criteria Decision Analysis" **OR** "multi criteria method" **OR** "cost effective* prioritization" **OR** "cost effective* prioritisation" **OR**"cost effective* analysis" **OR** "Spatial analysis" **AND** Marxan* **OR** MarCon* **OR** MarZone **OR** Spexan **OR** Spot **OR** Sites **OR** Zonation **OR** C-Plan **OR** CPLAN **OR** ResNet **OR** BioRap **OR** CREDOS **OR** MultCSync **OR** TARGET **OR** TRADER **OR** WorldMap **OR** prioritizR **OR** "Maritime Use Conflicts (MUC) Analysis" **OR** "Maritime Use Conflicts Analysis" **OR** "MarineMap" **OR** OceanMap **OR** CCRES **OR** CLUZ **OR** MinPatch **OR** "Zonae Cogito" **OR** "NatureServe Vista" **OR** PANDA **OR** SeaSketch **OR** PPGIS **OR** "Public Participation GIS" **OR** "Participatory GIS" **OR** PGIS **OR** GAMS **OR** CPLEX **OR** Gurobi **OR** "Integer Linear Program*" **OR** "integer programming" **OR** "multi-criteria" **OR** "multi-action" **OR** portfolio* **OR** "Decision Support System" **OR** DSS **OR** "decision support tools" **OR** DST **AND** "Protected area" **OR** "marine protected area" **OR** MPA **OR** "priority area" **OR** "area-based conservation" **OR** "conservation priorit*" **OR** "Management Area" **OR** "Nature Reserve" **OR** "Marine reserve" **OR** "National Park" **OR** Sanctuary **OR** "Marine Park" **AND** "Planning Unit*" **OR** "Analysis Unit*" **OR** cell **OR** hexagon* **OR** grid **OR** square* **OR** subcatchment* **OR** micro- catchment* **OR** "line segments" **OR** "linear segments" **OR** "linear units" **AND** marine **OR** sea* **OR** ocean* **OR** bay **OR** gulf **OR** coastal
**Results:**	142 documents
**Database 3**	**Scholar google**
**Date of** ** search:**	January 26, 2023
**Query:**	"systematic conservation plan*" (marine OR sea* OR ocean* OR bay OR gulf)
**Results:**	8000 results (only the first 200 will be included)

### Search strategy

The search strategy will apply a combination of keywords within documents’ title, abstract, and keywords using three different databases: The bibliographic search will be performed in three databases/ platforms, *i.e.*, 1) Web of Science – Core Collection, 2) Scopus and 3) Google Scholar (see Table 2 for the detailed combination used for each database). The total of the documents resulted from the search in Web of Science and Scopus will be considered for eligibility. Regarding Google Scholar database, only the first 200 hits will be considered ( Haddaway *et al.*, 2015).

### Study/source of evidence selection

Following the search, all identified citations will be collated, uploaded to the software Covidence and duplicates removed. Covidence is an online collaboration software platform designed to facilitate the process underpinning systematic and other literature reviews. Following the "team approach" recommended by Levac *et al.* (2010), titles and abstracts of each document will be initially screened by two independent reviewers for assessment against the inclusion criteria reported in the Table 1. Potentially relevant sources selected will be subsequently retrieved and uploaded in full. Thus, two independent reviewers will assess against the inclusion criteria the full-text of each document. Reasons for exclusion of sources at full-text that do not meet the inclusion criteria will be explicitly stated and reported in the ScR. Any disagreements that arise between the reviewers at each stage of the selection process will be resolved through the intervention of a third reviewer. The overall process with results about the excluded/included documents will be reported in the final ScR paper and presented using the PRISMA-ScR flow diagram ( Tricco *et al.*, 2018).

Covidence is a web-based collaboration software platform that streamlines the production of systematic and other literature reviews.

Studies already present in the systematic review by Álvarez-Romero *et al.* (2018) will be directly included and considered for the data extraction since it shares the same inclusion criteria with the present ScR.

### Data extraction

Two independent reviewers will extract data from the documents included in the ScR using a data extraction tool provided by Covidence and modified accordingly to the specific objectives of the ScR. Table 3 shows fields and information that have to be extracted from each paper. Any disagreements that arise between the reviewers will be resolved through the intervention of a third reviewer. If necessary, authors of papers will be contacted to request missing or additional data.

**Table 3.** Data extraction tool of the scoping review (ScR).

Field	Title	Answer	Question	Remarks/ guidance to reviewers (format, definitions, examples, etc.)
F1	**Author(s)**	free text	*Who are the authors of the* * document?*	Last name, first name
F2	**Title**	free text	*Which is the title of the document?*	Full title
F3	**Year of publication**	free text	*Which is the year of publication of* * the document?*	YYYY (4 digits) e.g., 2021
F4	**Journal**	free text	*Which is the title of the journal?*	Full-title (not abbreviated)
F5	**Keywords**	free text	*Which are the keywords of the* * document?*	-
F6	**DOI**	free text	*Which is the DOI of the document (if* * available)?*	Add DOI in the cases of peer- reviewed articles. E.g. 10.11124/ JBISRIR-D-19-00434
F7	**URL**	free text	*Which is the URL of the document (if * *available)?*	Add URL in the cases of grey literature retrieved via the internet (mandatory when DOI is not available).
F8	**Literature category**	peer-reviewed literature; grey literature	*Which is the literature category of* * the document?*	-
F9	**Literature type**	article; conference paper; organizational paper; report	*Which is the literature type of * *document?*	-
F10	**Literature source**	Scopus & Web of Science; Google Scholar; other	*Which is the source of literature?*	If the source of literature is not included in the predefined answers add “other”.
F11	**Language**	English; Spanish; German; Italian; Greek; French	*Which is the language of the* * document?*	-
F12	**Continent**	Africa; Antarctica; Asia; Europe; North America; Oceania/Australia; South America; more than one continent; global	*Which is the continent where the * *study takes place?*	-
F13	**Marine realm (sensu ** **Spalding *et al.*, 2007)**	Arctic; temperate northern Atlantic; temperate northern Pacific; tropical Atlantic; western Indo- Pacific; central Indo-Pacific; eastern Indo-Pacific; tropical eastern Pacific; temperate South America; temperate southern Africa; temperate Australasia; southern Ocean; more than one realm; global	*Which is the marine realm where the * *study takes place?*	-
F14	**Scale**	Global, Multi-national, National, Sub-national	*Which is the scale where the study* * takes place?*	-
F15	**Country**	Afghanistan, Albania, Algeria, Andorra, Angola, Antigua and Barbuda, Argentina, Armenia, Australia, Austria, Azerbaijan, Bahamas, Bahrain, Bangladesh, …	*Which is the country where the study* * is located?*	-
F16	**Rationale**	Arbitrary	*Which is the rationale for the* * Systematic Conservation Planning* * implementation?*	Mention (copy-paste from the manuscript) the specific rationale for the SCP implementation.
		Expert advice		
		Ecological requirements		
		Socioeconomic considerations		
		Legal mandate		
		National/international goals		
		Previous plan/study		
		Other (specify)		
F17	**Systematic ** **Conservation Planning** ** (SCP) extent**	free text (number)	*Which is the area of the potential* * SCP (in km ^2^)?*	Provide the area (in km ^2^) of the SCP
F18	**SCP contribution**	percentage	*What is the % contribution of the* * SCP in the country/study area*	Provide the % contribution that counts for the attainment of spatial targets
F19	**Methodology 1**	optimization algorithm; multi-criteria analysis; ranking; cost-benefit analysis; consensus; other	*Which is the DST used by the study?* * If other is selected it should be* * specified in the ‘comments’ field*	-
F20	**Methodology 2**	optimization algorithm; multi-criteria analysis; ranking; cost-benefit analysis; consensus; other	*Which is the DST used by the study?* * If other is selected it should be* * specified in the ‘comments’ field (if* * more than one DST was used)*	-
F21	**Optimization ** **algorithm**	Marxan; Marxan with Zones; Marxan Connect (or Marxan with Connectivity); Zonation; C-Plan; Multi-Link; Prioritize- R; other; none	*Which is the optimisation algorithm * *applied?*	-
F22	**Climate Change (CC) ** **resilience**	Yes/no	*Does the study aim to create MPA* * networks resilient to CC?*	-
F23	**CC refugia** ** identification**	Yes/no	*Were climatic refugia identified?*	-
F24	**CC refugia method**		*If yes, how were CC refugia* * identified?*	-
F25	**CC in Decision Support ** **Tool (DST)**	None (climate change was not incorporated)	*How was CC incorporated into the* * DST?*	-
		Design /size, spacing and replication)		
		Location (include/exclude sites)		
		Representation (adjusted)		
		Analyses of dynamics		
		Adaptive management		
		Other (please specify)		
F26	**Habitat types**	Habitat types (Benthic)	*Which marine species / habitats * *were targeted in the SCP?*	-
		Habitat types (Pelagic)		
F27	**Connectivity of sp./** **habitats**	Yes/no	*Was connectivity of species (sp.) or* * habitats accounted for?*	-
F28	**Type of connectivity** ** for sp./habitats**	Behaviour - adults	*What type of connectivity for * *species/habitats was included in the* * analysis?*	-
		Behaviour - larvae		
		Dispersal - adults		
		Dispersal - larvae		
		Genetics		
		Habitat quality		
		Ocean currents		
		Network topology		
		Other (please specify)		
F29	**Threats included**	Yes/no	*Were any type of threats included in* * the analysis (e.g., fishing pressure, * *human footprint)?*	-
F30	**Connectivity of threats**	Yes/no	*Was connectivity of threats * *accounted for?*	-
F31	**Type of connectivity** ** for threats**	Structural; ONLY basic lateral	*What type of connectivity for threats * *was included in the analysis?*	-
F32	**Land-sea planning**	Yes/no	*Was this case study an integration of* * land-freshwater-marine planning?*	Marine-freshwater OR marine- terrestrial OR all three realms
F33	**Inter-realm** ** connectivity**	Yes/no	*Was inter-realm connectivity* * accounted for?*	-
F34	**Cumulative Effects ** **Assessments (CEA)**	Yes/no	*Were CEA accounted for in the * *analysis?*	-
F35	**CEA method**		*If yes, what method was used to * *account for CEA in SCP?*	-
F36	**Other Effective** ** Conservation Measures** ** (OECMs)**	Yes/no	*Were OECMs included in the* * planning?*	-
F37	**Type of OECMs**		*If yes, what types of OECMs were* * accounted for?*	e.g., FRAs, archaeological sites
F38	**OECMs method**		*What method was used to include * *OECMs in SCP?*	-
F39	**Restoration ** **prioritization**	Yes/no	*Were sites for restoration (besides* * protection) prioritized?*	-
F40	**Restoration method**		*What method was used to prioritize* * restoration sites?*	-
F41	**Invasive Alien Species** ** (IAS) of concern**	Yes/no	*Were IAS considered?*	-
F42	**IAS in planning**	Ignore/do not care; avoid; protect	*Which approach for IAS in planning* * was applied?*	-
F43	**IAS planning method**	free text	*If avoid or protect, what specific* * method was used to include them * *in SCP?*	-
F44	**Socioeconomic and ** **cultural objectives**	Yes/no	*Were socioeconomic or cultural * *objectives included in SCP?*	-
F45	**Socioeconomic and** ** cultural objectives ** **- methods**	free text	*How were socioeconomic and * *cultural objectives included in SCP?*	-
F46	**Tradeoffs**	Yes/no	*Were ecological and socioeconomic/* *cultural tradeoffs accounted for?*	-
F47	**Tradeoffs methods**	free text	*What methods were applied* * to account for ecological and* * socioeconomic/cultural tradeoffs?*	-
F48	**Gaps of knowledge**	free text	*Which are the gaps of knowledge* * identified by the study (if any)?*	Briefly describe the gaps of knowledge identified by the study.
F49	**Policy** ** recommendations**	free text	*Which are the policy* * recommendations proposed by the* * study (if any)?*	Briefly describe the policy recommendations proposed by the study.
F50	**Stage of SCP**	proposed, committed, designated, implemented, target reached	*At which stage the SCP is?*	

### Data analysis and presentation

The evidence synthesised by the ScR will address all the review objectives and questions. Data will be presented graphically and in a diagrammatic/tabular form. A narrative summary will accompany the tabulated and charted results and will describe how the results relate to the review’s objective and questions. In particular, the following outputs, based on a quantitative synthesis of the data, will be presented: i) A global map showing the amount of case studies for each country; ii) plots showing the criteria used in performing SCP across all the case studies; iii) meta-analyses to assess the correlation between the criteria used and the SCP outcomes/implementation stage across case studies; iv) meta-analyses to assess the correlation between the SCP spatial contribution and the SCP outcomes/implementation stage across case studies; and v) mapping of network for keywords, data gaps, policy recommendations, and good practices across case studies.

## Study status

The study is currently at the end of the data extraction step. The next stages will be as follows: i) the integration of data with those coming from the dataset published by Álvarez-Romero *et al.* (2018); ii) data analysis and presentation; and iii) ScR preparation and submission for publication.

## Ethics and consent

Ethical approval and consent were not required.

## Data Availability

No data are associated with this article.
