# Supplementary material for: Current practices in marine systematic conservation planning: protocol for a global scoping review
Source: Open Res Eur. 2023 Sep 5;3:136. [Version 1] doi: 10.12688/openreseurope.16300.1 (PMC10858334; doi:10.12688/openreseurope.16300.1)
Supplement: Peer review history

# Reviewer report: Reviewer response for version 1

**DOI:** 10.21956/openreseurope.17597.r37979

**Published:** 2024-02-09

**Authors:** Cinzia Podda

**Affiliations:** 1 International Marine Centre, Oristano, Italy

A protocol for a global scoping review of marine systematic conservation planning for designing MPAs networks is here described.

 The protocol is presented in a linear and readable manner and I recognize the importance of the effort of such work as an attempt to standardize and aggregate all existing global information in the context of systematic marine conservation planning.

 I also recognize the ambition of this work, above all in wanting to investigate very complex and crucial questions (connectivity, alien species, among others), all issues still little addressed with an integrated planning approach. I presume that this is the reason why a limited number of works has been obtained in the scoping review in Scopus and WoS.

 I would further emphasize the importance of having dealt critical points (such us ecological processes, balancing conservation, socio-economic, and cultural objectives, existing guidelines for improved conservation planning) that are rarely considered or otherwise treated in a disgregated way.

 Tables are very useful and well summarized, despite the amount of content. Just a small specific comment relating to table 3, Field28 Type of connectivity, it might be appropriate, for better clarity of the outputs, to consider adult movements or migration (rather than dispersal). I also suggest considering the vertical connectivity between the typologies of connectivity for a more detailed suite of options.

 About marine connectivity I bring to your attention the following review: https://www.frontiersin.org/articles/10.3389/fmars.2023.1271397/full

 I agree with the fact that often the tools for identifying and prioritizing areas to be protected are only mentioned but not expressly rigorously described, this in some way tends to limit the application of shared approaches on a global scale.

 Finally, for future implications of this research, from a planning perspective, it would be interesting to quantify (in %, in km ^2^ or in hectares) the extension of the areas currently subjected to SCP as well as their mapping.

Is the study design appropriate for the research question?

Yes

Is the rationale for, and objectives of, the study clearly described?

Yes

Are sufficient details of the methods provided to allow replication by others?

Yes

Are the datasets clearly presented in a useable and accessible format?

Yes

Reviewer Expertise:

Aquatic ecology and maritime spatial planning, focusing on ecological modeling and ecological connectivity

I confirm that I have read this submission and believe that I have an appropriate level of expertise to confirm that it is of an acceptable scientific standard.

## References

[1] : Marine spatial planning for connectivity and conservation through ecological corridors between marine protected areas and other effective area-based conservation measures. *Frontiers in Marine Science* .2023;10: 10.3389/fmars.2023.1271397 10.3389/fmars.2023.1271397

# Reviewer report: Reviewer response for version 1

**DOI:** 10.21956/openreseurope.17597.r35477

**Published:** 2023-11-27

**Authors:** Hong Ching Goh

**Affiliations:** 1 Department of Urban and Regional Planning, Faculty of Built Environment, University of Malaya, Kuala Lumpur, Malaysia

This is an easy reading piece, and the manuscript has been arranged with straightforward points in logical sequence and easy to follow. I recognize that this is an ambitious attempt and is possibly embedded within a larger research program and the exercise will be very much dependent on the resources made available. Couples of suggestions/comments that I have for this manuscript are as follows. Although some suggestions refer to specific tables, it is meant to draw a larger attention to the overall consideration of the protocol.

- In Table 2 which spell out the details of scoping review search strategy per database, *i.e.*, name of database, date of search, search query, and results (as the number of documents returned by the search), in the category of Marine Park, Marine Management Plan, whether transboundary MPA are considered. If applicable, suggest including the term transboundary conservation.  
- In Table 3 Data extraction tool of the scoping review (ScR), I would like to suggest the authors to include Southeast Asia as a separate (sub)category considering its geographical uniqueness (and the diversities) especially the oceans, the length of coastlines and the number of islands that this region covers.
- As one of the outputs will be ‘A global map showing the amount of case studies for each country', one concern I have is the language barrier for countries such as China and Indonesia, where policy documents and ‘blue papers’ might be available only in the national language, which could lead to exclusion from this scoping.
- The consideration of having cultural and/or intangible values either within the context of ecosystem services or beyond, can be more explicitly mentioned in the review question #2 since cultural objective is highlighted.
- It is also worth considering whether SCPs include provisions for monitoring, evaluation and potential revision to existing planning.
- A reference that may be deemed useful is linked.

Is the study design appropriate for the research question?

Yes

Is the rationale for, and objectives of, the study clearly described?

Yes

Are sufficient details of the methods provided to allow replication by others?

Yes

Are the datasets clearly presented in a useable and accessible format?

Yes

Reviewer Expertise:

Coastal planning with social science perspective focusing on human-environment interaction

I confirm that I have read this submission and believe that I have an appropriate level of expertise to confirm that it is of an acceptable scientific standard.
